# Food safety practice and its associated factors among food handlers in food establishments of Mettu and Bedelle towns, Southwest Ethiopia, 2022

**DOI:** 10.1186/s40795-022-00651-3

**Published:** 2022-12-22

**Authors:** Sanbato Tamiru, Kebebe Bidira, Tesema Moges, Milkias Dugasa, Bonsa Amsalu, Wubishet Gezimu

**Affiliations:** 1 Department of Nursing, Mettu University, Mettu, Ethiopia; 2 Department of Public Health, Mettu University, Mettu, Ethiopia

**Keywords:** Food safety, Practice, Food handlers

## Abstract

**Background:**

Food safety and hygiene are currently a global health concern, especially in unindustrialized countries, as a result of increasing food-borne diseases (FBDs) and accompanying deaths. It has continued to be a critical problem for people, food companies, and food control officials in developed and developing nations.

**Objective:**

The objective of the study was to assess food safety practices and associated factors among food handlers in food establishments in Mettu and Bedelle towns, south-west Ethiopia, 2022.

**Methods:**

A community-based cross-sectional study was conducted from February to March 2022, among 450 randomly selected food handlers working in food and drink establishments in Mettu and Bedelle towns, Southwest Ethiopia. Data was collected using an interviewer-administered structured questionnaire. The data was coded and entered into Epi Data version 3.1 before being exported to SPSS version 20 for analysis. Both bivariate and multivariable logistic regression models were fitted. An adjusted odds ratio and a 95% confidence level were estimated to assess the significance of associations. A *p*-value of < 0.05 was considered sufficient to declare the statistical significance of variables in the final model.

**Result:**

A total of 450 food handlers participated in the study, making the response rate 99.3%. About 202 (44.9%) of respondents had poor practices in food safety. Lack of supervision (AOR = 6.2, 95% CI: 3.37, 11.39), absence of regular medical checkups (AOR = 1.98; 95% CI: 1.14, 3.43), lack of knowledge of food safety practices (AOR =2.32; 95% CI: 1.38, 3.89), availability of water storage equipment (AOR =0.37; CI: 0.21, 0.64), and unavailability of a refrigerator (AOR =0.24; 95% CI: 0.12) were factors significantly associated with food safety practices.

**Conclusion:**

The level of poor food safety practices was remarkably high. Knowledge of food safety, medical checkups, service year as food handler, availability of water storage equipment, availability of refrigerator, and sanitary supervision were all significantly associated with food safety practice. Hence, great efforts are needed to improve food safety practices, and awareness should be created for food handlers on food safety.

**Supplementary Information:**

The online version contains supplementary material available at 10.1186/s40795-022-00651-3.

## Introduction

Food safety is described as the circumstance and control required to ensure the safety, wholesomeness, and suitability of the food during its production and consumption. It is the primary public health concern for many countries, which is essential to prevent foodborne illness and enhance the well-being of humans [1, 2]. Food safety and hygiene are currently a global health concern, particularly in developing countries, as a result of increasing food-borne diseases (FBDs) and accompanying deaths, and they also continue to be a critical problem in developed and developing nations for people, food companies, and food control officials [2].

Food-borne diseases (FBD) are associated with outbreaks, threaten global public health security, and have become an international concern as a growing public health issue [3]. According to the World Health Organization (WHO), FBDs in developing nations are serious because of bad hygienic food handling methods, poor understanding, and the absence of infrastructure. This is the result of poor use of food handling and sanitation practices, inadequate food safety laws, weak regulatory systems, and a lack of financial resources [4, 5].

Findings of different studies showed that there are relatively few food safety problems in some Asian countries like Indonesia, Jordan, and Saudi Arabia, which ranged from 10 to 19.31%, respectively [6, 7]. But when we look at the findings of the study conducted in Malaysia, the magnitude of poor food safety practices was about 41.7% [1]. A study conducted in some parts of the country revealed that there is a high magnitude of food safety problems in food and drink establishment centers, ranging from 46.3 to 72.67% [3, 8, 9].

WHO disclosed that 1 in 10 individuals worldwide is sick from foodborne illnesses secondary to unsafe food practices and the use of contaminated foods [10]. Food safety practices are worse in developing countries, including Ethiopia; according to previous studies conducted in Addis Ababa and other areas of the country, less than half of food handlers have maintained satisfactory safety practices in handling food in the studied food establishments [3, 8, 9].

Foodborne diseases are prevalent in Ethiopia; the country’s annual incidence of foodborne illnesses ranged from 3.4 to 9.3% [3]. Food safety practices also have economic implications. The effects of food-related illness expenditures on hospital treatments are about US$ 110 billion annually in developing countries, which results in decreased production [11]. Several factors, like prevailing poor food handling and sanitation practices, inadequate food safety training, weak regulatory systems, a lack of financial resources, low educational status, and a lack of knowledge, have been identified as affecting food safety [12].

Efforts have been made globally by preparing a food safety guideline with the help of the World Health Organization. Similarly, in Ethiopia, significant work has been done with regards to food safety by preparing a national food safety policy and guidelines and assigning a regulatory department in the health office at different levels, though people are still suffering from morbidities and mortalities related to food-borne diseases. This is mainly attributed to food safety practices [3, 13]. Only a few studies have been conducted in Ethiopia with regard to food safety practices. Hence, the purpose of this study was to assess food safety practices and associated factors among food handlers in the food establishment centers of Mettu and Bedelle towns.

## Methods

### Study design, period, and area

A community-based cross-sectional study was conducted from February 21 to March 21, 2022, in Mettu and Bedelle towns. The two towns are located in the Oromia Regional State of southwest Ethiopia. Mettu is the capital of the Ilubabor Zone, and Bedelle is the capital of the Buno Bedelle Zone. Mettu and Bedelle towns are located about 600 and 480 km southwest of Addis Ababa, respectively. The two towns are home to different institutions and factories like Mettu University, Mettu Karl Comprehensive Specialized Hospital, Bedelle General Hospital, Bedelle Brewery Factory that provide services for a large number people in the southwest region of the country. There were 188 food establishments and 1015 food handlers in the study area, according to data gathered from the trade and industry bureaus of the two towns.

### Populations and eligibility criteria

All food handlers who worked in food and drink establishments in Mettu and Bedelle towns were considered the source population, whereas all selected food handlers who worked in selected food and drink establishments in the two towns were study participants in this study.

Food handlers working in preparation, cleaning, and service areas of food establishments at the time of the study were included in the study. However, food handlers who were not available during the data collection period and who could not give a response due to severe illness were excluded from the study.

### Sample size determination and sampling procedure

The sample size was determined using a single population proportion formula:

$$n={\left(z\frac{\alpha }{2}\right)}^2\frac{(pq)}{d^2}$$

Where Z = 1.96, the confidence limits of the survey result (value of Z at α/2 or critical value for normal distribution at 95% confidence interval).

*p* = 0.5 (50%), the population proportion of food safety practices from study conducted in Fiche town [5].

d = 0.05, the desired precision of the estimate

q = 1-p

So the calculated sample size was, $n={(1.96)}^2\frac{\left(0.5\ast 0.5\right)}{0.05^2}=384$

Since the total number of food handlers in the study area was less than 10,000, we have utilized correction formula, that gives nf =288. After adding a 5% non-response rate and a design effect of 1.5, the final sample size of 453 was used for this study.

The list of existing food establishments and the number of food handlers currently working in food establishments were obtained from Mettu and Bedelle towns’ Trade and Industry Office. Then, food establishments for this study were randomly selected from a total list of food establishments. Next, study participants were proportionately allocated to each selected food establishment based on the number of food handlers. Then, an updated list of food handlers was taken from the manager or owner of the selected establishment. Finally, study participants were selected using a simple random method from each establishment.

### Study variables

The dependent variable of this study was food safety practice, and the independent variable includes socio-demographic factors (educational level, age, gender, marital status, and work experience), institutional factors (training, supervision, and availability of guidelines for food safety), health-related factors (medical check-ups and sick leave during illness), knowledge-related factors (knowledge of methods to prevent contamination and knowledge of food safety practices), and sanitary facility-related factors (three-compartment dishwashing systems, refrigerators in the kitchen, and water supply).

### Data collection tools and procedure

Data were collected using an interviewer-administered standardized questionnaire adapted and modified from previously published studies [3, 8, 9, 13]]. The questionnaire was structured into six parts: socio-demographic parts with six questions, food safety knowledge with nine questions, basic sanitary facilities with seven questions, institutional factors with four questions, health-related with two questions, and food safety practice with twelve questions.

Food safety knowledge was assessed using nine closed-ended questions with two possible answers: “yes” or “no.” The questions mainly focus on the personal hygiene of food handlers, temperature control, cross-contamination, food storage, and equipment hygiene. In assessing knowledge, one score was given for every correct answer and zero score for incorrect answers or unanswered questions. Then, the responses to these questions were added together to generate a knowledge score. Food handlers who obtained a total score greater than the mean value were considered to have good food safety knowledge, and those who had scores less than the mean value were considered to have “poor food safety knowledge.”

Food safety practices were also assessed using 12 closed-ended questions with two possible answers: “yes” or “no.” One score was given for every standard practice and zero for every unsafe practice. Food handlers with a total score greater than the mean were considered to have “good food safety practices,” while those with a score less than the mean were considered to have “poor food safety practices.” The data was collected by three diploma nurses, and the overall data collection processes were supervised by one health officer after two days of training.

### Data quality assurance

The quality of the data was ensured through all data collection tools and was translated into the local language and back-translated to English by language experts to ensure its consistency. Training of data collectors and supervisors was conducted to enable them to acquire the basic skills necessary for data collection and supervision, respectively. A pre-test was done on 5% of the sample in Gore town, and based on the results of the pre-test, necessary modifications were made. After data collection, the completeness of the data was checked by the principal investigator ahead of data entry. Incomplete and inconsistent questionnaires were excluded from the analysis.

## Data analysis

The collected the data was curated according to the study objectives. The coded responses were entered into EpiData and exported to SPSS for analysis. A descriptive analysis was used to describe the percentages and number of distributions of the respondents by socio-demographic characteristics and other relevant variables in the study. A binary logistic regression analysis was performed on the independent variables and their proportions, and a crude odds ratio was computed against the outcome variable. The independent variables with a *p*-value less than 0.25 were entered into the multivariable logistic regression analysis to control for potential confounders and identify significant factors associated with outcome variables. Finally, a *p*-value of less than 0.05 at the 95% CI was used to claim statistical significance.

### Ethics and approval processes

The ethical clearance letter was obtained from ethical committee of Mettu University, college of health science before conducting study. After the interviewer read and clearly explained the study’s benefits and risks, a written consent was obtained from the study participants. Then literate participants signed, whereas uneducated participants put their fingerprints on the consent form to shown their willingness to participate. Confidentiality of the data was maintained at all times.

## Results

### Socio-demographic characteristics of food handlers

In this study, a total of 450 food handlers participated out of a total of 453 with a response rate of 99.3%. The mean age of the participants was 29.3 years (SD = 5.28). The majority (54%) of food handlers were female. One hundred ninety-one (42.4%) of them attended secondary education, while only nine (2%) of them had least a higher education qualification. More than half (62.2%) of food handlers were married. Regarding service year as food handler, about 181 (40.2%) of respondents had worked for 2–4 years [Table 1].

**Table 1:** Socio-demographic characteristics of food handlers in food establishments of Mettu and Bedelle Towns, southwest Ethiopia, 2022 (*n* = 450)

Characteristics	Frequency	Percentage
**Sex**		
Male	207	46
Female	243	54
**Age**		
< =20 years	11	2.4
21-30 years	240	53.3
31-40 years	185	41.1
> =40 years	14	3.1
**Educational status**		
Unable to read and write	55	12.2
Primary education	138	30.7
Secondary education	191	42.4
College Diploma	57	12.7
Degree and above	9	2
**Marital status**		
Married	280	62.2
Unmarried	136	30.2
Divorced	17	3.8
Widowed	17	3.8
**Service year**		
< 2 years	101	22.4
2-4 years	181	40.2
5-7 years	115	25.6
8-10 years	39	8.7
> 10 years	14	3.1
**Responsibility**		
Main chef/cook	165	36.7
Assistant chef/cook	65	14.4
Waiter	220	48.9

### Practice of food handlers on food safety

Among the 450 total food handlers, 417 (92.7%) of them were not checking the temperature of food. The majority, 333 (74%) of food handlers, did not wash their hands after sneezing. Two hundred and eleven (46.7%) participants did not wear hair covers. About 10.2% of food handlers did not trim their fingernails. Majority, 387(86%) of the participants, did wash their hands after touching unwrapped food, whereas 366 (81.3) of them wash their hands before touching cooked food. Moreover, 145 (32.2%) of participants did not use separate utensils for raw and cooked foods [Table 2].

**Table 2:** Food handling practices among food Handlers in food establishments of Mettu and Bedelle Towns, Southwest Ethiopia, 2022 (*n* = 450)

Characteristics	Frequency	Percentage
Wash hand before touching cooked food		
No	84	18.7
Yes	366	81.3
Wash hand after touching unwrapped food		
No	63	14
Yes	387	86
Use separate utensils for raw and cooked food		
No	145	32.2
Yes	305	67.8
Thaw frozen food at room T^o^		
No	192	42.7
Yes	258	57.3
Check expire date of Commercial product / foods		
No	180	40
Yes	270	60
Check T^o^ of food		
No	417	92.7
Yes	33	7.3
Use glove before touching foods		
No	188	41.8
Yes	262	58.2
Wash hand before using glove		
No	28	6.2
Yes	236	52.4
Wash hands after using glove		
No	29	6.4
Yes	235	52.2
Shorten/trim finger nails		
No	46	10.2
Yes	404	89.8
Use hair cover/cape		
No	211	46.9
Yes	239	53.1

Sanitize/wash hands after sneezing (before touching food)		
No	333	74
Yes	117	26

### Level of food safety practice

**Fig. 1:**
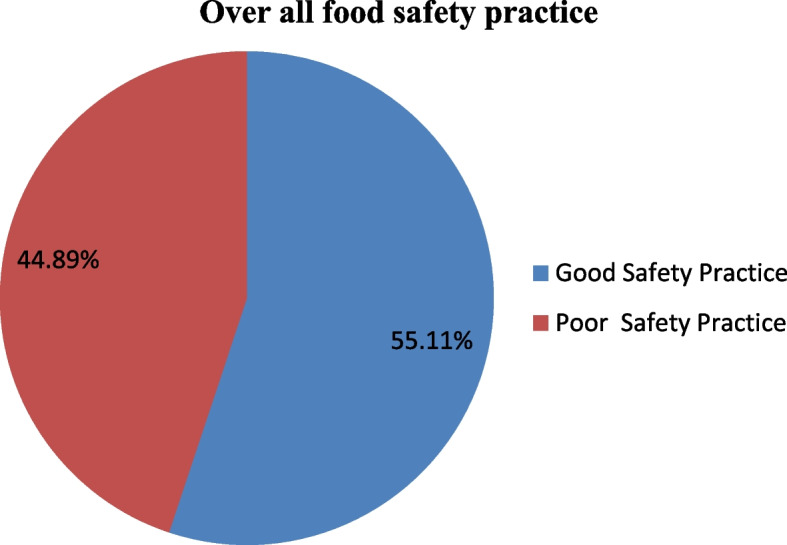
Overall food safety practice of food handlers working in food and drink establishment in Mettu & Bedelle towns south west Ethiopia, 2022 (*n* = 450)

In general, out of all participants, 202 (44.89%) had poor food safety practices, while 248 (55.1%) had good food safety practices [Fig. 1].

### Factors associated with food safety practice

In the bivariate analysis, variables like sanitary inspection, medical checkup, food safety knowledge, the presence of a refrigerator, the service year as a food handler, the presence of guidelines or guiding instructions, and the unavailability of water storage equipment were shown to be associated with the outcome variable. In the multivariable logistic analysis, the variables sanitary inspection (AOR = 6.2; 95% CI: 3.37, 11.39), medical checkup (AOR = 1.98; 95% CI: 1.14, 3.43), knowledge of food safety practices (AOR = 2.32; 95% CI: 1.38, 3.89), the presence of a refrigerator (AOR = 0.24; CI: 0.12, 0.45), and availability of water storage equipment (AOR = 0.37; CI: 0.12, 0.45) were found to be significantly associated with food safety practices.

The study revealed that food handlers with a poor level of knowledge were 2.32 times more likely to have poor food safety practices than those with a good level of knowledge (AOR = 2.32; 95% CI: 1.38, 3.89). The likelihood of having poor food safety practices among food handlers who did not have regular medical checkups was nearly two times higher than among food handlers who have regular medical checkups (AOR = 1.98; 95% CI: 1.14, 3.43). Moreover, poor food safety practice was 6.2 times higher among non-supervised food handlers as compared to their counterparts (AOR = 6.2; 95% CI: 3.37, 11.39) [Table 3].

**Table 3:** Bivariate and multivariate logistic regression analysis output of factors associated with food safety practices among food handlers in food establishments of Mettu and Bedelle Towns, southwest Ethiopia, 2022 (*n* = 450)

Variables	Food safety practice		COR(95%CI)	AOR(95%CI)
	Poor	Good		
**Sanitary inspection**				
**No**	166	126	4.46(2.88,6.91)	6.2(3.37,11.39)***
**Yes**	36	122	Ref	
**Medical checkup**				
**No**	121	124	1.49(1.02,2.17)	1.98(1.14,3.43)*
**Yes**	81	124	Ref	
**Food safety knowledge**				
**Poor**	134	91	3.4(2.30,5.01)	2.32(1.38,3.89)**
**Good**	68	157	Ref	
**Refrigerator in the kitchen**				
**No**	113	221	0.155(0.09,0.25)	0.24(0.12,0.45)***
**Yes**	89	27	Ref	
**Service year as food handler**	15	86	Ref	
**< 2 years**	60	121	0.29(0.006,0.14)	0.032(0.005,0.18)***
**2–4 years**				
**5–7 years**	82	33	0.083(0.018,0.38)	0.115(0.021,0.618)*
**8–10 years**	33	6	0.414(0.088,1.95)	0.46(0.085,2.51)
**> 10 years**				
	12	2	0.917(0.16,5.17)	
**Presence of guiding rule**				
**No**	116	200	0.32(0.21,0.49)	0.74(0.42,1.33)
**Yes**	86	48	Ref	
**Water storage equipment**				
**No**	60	118	0.46(0.31,0.68)	0.37(0.21,0.64)***
**Yes**	142	130	Ref	

Notes: * significant at *p*-value < 0.05, ** significant at p-value < 0.01, ***significant at *p*-value < 0.001

Abbreviations, AOR adjusted odds ratio, COR crude odds ratio

## Discussion

Ensuring optimal food safety practices is still a major global challenge, particularly in developing countries like Ethiopia. This has in turn resulted in a high prevalence of FBD [3]. This study aimed to assess food safety practices and its associated factors among food handlers in food establishments of Mettu and Bedelle towns, Southwest Ethiopia.

The findings of the present study showed that 44.9% (CI: 40.29, 49.49) of participants had poor food safety practices. This finding is higher than studies conducted in Indonesia (10%) [2], Saudi Arabia (19.3%) [6], Nigeria (30.5%), [14], Arba Minch town, Southern Ethiopia (32.6%) [15], Dessie town, Northern Ethiopia (28%) [16], and Assosa Western Ethiopia [17]]. The variation might be due to differences in study settings and food handler’s socio-demographic profile. But it was lower than studies conducted in Fiche (50%) [3], and Gondar town (53.3%) [12]. The possible reason for discrepancies might be the difference in the study design, cutoff points, and year of study. However, the present finding was comparable with studies conducted in Debra Markos town (46.3%) [9], Woldia town, Northeast Ethiopia(46.5) [18], Dangila town, North West Ethiopia(47.5%) [19] and Batu town Central Ethiopia [20].

Regarding factors associated with poor food safety practice, this study revealed that; a lack of regular medical checkup was significantly associated with poor food safety practices. The likelihood of having poor food safety practices among food handlers who did not have regular medical checkups was nearly two times higher than that of those food handlers who have regular medical checkups. This finding was supported by a study conducted in Fiche, Gondar towns, and Dessie town [3, 12, 17]. This might be due to behavioral change following counseling given during a medical checkup.

The study revealed that sanitary inspection is significantly associated with food safety practice. Poor food safety practice was 6.2 times higher among non-supervised food handlers as compared to their counterparts. This finding is supported by a previous study conducted in Arba Minch town of Southern Ethiopia [15].

In the present study, knowledge of food safety was significantly associated with food safety practices. Food handlers with a poor level of food safety knowledge were 2.33 times more likely to have poor food safety practices than those with a good level of knowledge. This study is supported by cross-sectional studies conducted in Gondar city, Debra Marcos town, Dangila town, Northern Ethiopia, and Batu town, Central Ethiopia [9, 12, 19, 20].

There was a significant association between food safety practices and sanitary inspection. The probability of having poor food safety practices was higher among food handlers who were not supervised than their counterparts. The present finding was supported by a study conducted in Assosa and Gondar city, Woldia town [12, 17, 18]. This might be due to the effect of advice and feedback given to supervised food handlers, managers, and the owners during an inspection.

The probability of having poor safety practices was 62.7% less likely among food handlers working in establishments having water storage equipment as compared to their counterparts. This might be due to easy access of water to cleansing. This finding was supported by a community based cross sectional study conducted in the Bole sub-city of Addis Ababa[ [8].

Moreover, the service year as a food handler was also significantly associated with food safety practices. The probability of having poor food safety practices among food handlers with 2–4 and 5–7 years of service was 96.8 and 88.5% less likely, respectively, as compared to those food handlers with a service year of less than 2 years. The finding was supported by a study conducted in Fiche and Debra Marcos town [3, 9]. This might be due to the positive effect of adaptation to a specific working environment and sharing experience from coworkers.

### Limitations of the study

The study was based on reported rather than observed practices related to food safety. Therefore, there was a risk that respondents may report what was expected of them but practice may be different. In addition, lack of universal consensus on the definition of good or poor practice was a challenge in the study. Furthermore, parasitic and microbiological laboratory investigations were not considered in this study.

## Conclusion

The level of poor food safety practices was remarkably high in the study area. Almost all food handlers did not use thermometers to check the temperature of the food. More than one-third of them were not using separate utensils for raw and cooked foods. Nearly half of food handlers did not use hair covers, and three-fourths of them did not practice sanitizing or washing their hands after sneezing prior to touching foods. Generally, there is an increased risk of FBD in association with the identified poor food safety practices. Factors like sanitary inspection, medical checkup, food safety knowledge, availability of refrigerator, service year as food handlers, and availability of water storage were identified as having significant associations with the identified poor level of food safety practice. Therefore, there is need to invest much more in food safety practices, and special emphasis should be given to food safety in order to reduce the risk of FBD and ensure optimal food safety practices.

### Recommendation

To improve food safety practices, all concerned bodies should play their roles. Food handlers should have regular medical checkups, maintain good hygiene, try to improve their knowledge and practice of food safety, and play a crucial role in ensuring good food safety practices. Food handlers should also use separate utensils for raw and cooked foods to reduce cross-contamination. Food establishment owners should avail themselves of equipment like refrigerators and water storage that can help ensure food safety by preventing spoilage and contamination.

Healthcare professionals and food professionals in collaboration need to conduct on-site supervision, inspect the hygiene of food handlers, and observe the way they are working towards food safety practices. They should conduct strict sanitary supervision on a regular basis and take timely corrective action (reward compliant food handler or constrain non-compliant handlers). In addition, they need to arrange for regular medical checkup of food handlers in collaboration with nearby medical facilities.

The trade and industry office should work in collaboration with health office and take food safety practices into consideration during the renewal of licenses of establishments, and the government or policy makers should enforce the implementation of HACCP as guiding instructions in all establishments as a mandatory requirement. Based on current findings, future researchers can conduct detailed investigations that are supported by microbial analysis and try to show a new approach to improving food safety practice.

### Ethics statements

The study was carried out in accordance with the principles of the Declaration of Helsinki. The study was conducted after getting ethical approval and clearance from the institutional review board (IRB) of Mettu University. A supportive letter was taken from Mettu University and submitted to each hospital, and permission was obtained from each hospital. Participation was completely voluntary, with no economic or other motivation, and informed consent was obtained from the study participants. All participants were informed of the study’s purpose and given the right to respond fully or partially to the questionnaire. They also had the right to withdraw at any time. Furthermore, Participants who agreed to participate in the study were asked to sign informed consent forms. The privacy and identity of participants were protected, and participants’ confidentiality was also assured by omitting their names from the informed consent form.

## Conflict of interest

The authors declare that they have no competing interests.

## Supplementary Information

**Additional file 1.** Annexe-1 Questionnaires and Consent forme.

## Data Availability

All the data generated or analyzed during the current study are available from the corresponding author upon reasonable request.
