# Supplementary material for: Food safety practice and its associated factors among food handlers in food establishments of Mettu and Bedelle towns, Southwest Ethiopia, 2022
Source: BMC Nutr. 2022 Dec 22;8:151. doi: 10.1186/s40795-022-00651-3 (PMC9773440; doi:10.1186/s40795-022-00651-3)
Supplement: Supplementary file 1 — Additional file 1. Annexe-1 Questionnaires and Consent forme. [file 40795_2022_651_MOESM1_ESM.docx]

# Annexe-1 Questionnaires and Consent forme

**1. Information Sheet**

Greeting Good morning / afternoon

My name is _____________. I’m data collectors of a survey on assessment food safety practice and its associated factors among food handlers of establishments in Mettu and Bedelle town. The information I collect will help the town and government at large to plan health services. Now you are randomly selected for the survey. The questions usually take about 20 to 25 minutes.

**The objective of the study:** To assess food safety practice and its associated factors among food handlers of establishments in Mettu and Bedelle town, south west; Ethiopia.

**The benefit of the study:** there is no direct benefit of the participant of the study. However theresults of this study will help in identifying problems related to food safety and contributes on input in considering a convenient programmaticapproach to solve the problem. The result of the study will be disseminated to concerned bodies, including to Mettu and BedelleHealth office.

**The risk of the study:** Participating in this study will not have any risk or harm.

**Right of participants:** you have full right either to participate or decline participation in this studyas a participant. You may respond to all the questions or you may not answer to the questions youdon’t want and you may end the interview at any time you want. You can ask any questions whichis not clean for you.

**Confidentiality:** any information forwarded will be kept confidential and names will not be writtenor specified.

**II. Informed consent**

As to the information given ahead, participating in this study has no any risk. Your names will not bewritten on this form and the information you give will never be shared to others. You may notanswer any questions that you don’t want to answer and you may end this interview anytimeyouwant. Now Iwould like to tell you that you are selected randomly to be participant of this study, your genuine response to interviews will be very important for the purpose of the study. At thesame time I would like to appreciate your voluntary participation in the survey after a thoroughunderstanding of the information given to you.

I have read this form or it has been read to me in the language I comprehend and understand all condition stated above.

Are you willing to participate in this study?

1. No (say thank you) 2. Yes (continue interviewing

Name of the principal investigators: ________________________

Name of interviewer_________________

Data of interviewer (Ethiopia calendar) ____/______/

Result of interview: 1. Completed 2. Refused3. Partial completed 4. Respondent not available

Cheeked by supervisor

Name___________________ Signature ______________. Date_______________

**English version questionnaire**

Instruction: circle the response from the alternative and write the answer for open ended on the space provided.

**Part I- Socio Demographic information**

| S/N | Questions | Choice of response | Code | Skip |
| --- | --- | --- | --- | --- |
|  | Sex | 1. Male 2. Female |  |  |
|  | How old are you? | _______ Age in complete year |  |  |
|  | What is your current marital status? | 1. Married 2. Single 3. Divorce 4. Widowed 5. Other (specify)----- |  |  |
|  | What is your educational status? | 1. un able to read and write 2. Primary education 3. Secondary education 4. college diploma 5. Degree or more |  |  |
|  | What is your responsibility in this organization? | 1. Main Chef 2. assistant Chef 3. waiter 4. Other specify ------ |  |  |
|  | How many Work experience /service year do you have? | 1. <2 Years 2. 2-4 Years 3. 5-7 Years 4. 8-10 Years 5. >10 Years |  |  |

**Part II. Food safety practice questions**

| S/N | Questions | Choices of response | Code | Skip |
| --- | --- | --- | --- | --- |
| 201 | Do you wash your hands after touching Un wrapped raw foods? | 1. Yes  2. No |  |  |
| 202 | Do you wash your hands before touching cooked Foods? | 1. Yes  2. No |  |  |
|  | Do you use separate utensils when preparing raw and cooked foods? | 1. Yes  2. No |  |  |
| 204 | Do you thaw frozen foods at room temperature? | 1. Yes  2. No |  |  |
| 205 | Do you check the expiry dates of commercial products before use? | 1. Yes  2. No |  |  |
|  | Do you check the temperature of food? | 1. Yes  2. No |  |  |
|  | Do you use gloves when serving unwrapped food? | 1. Yes  2. No |  |  |
|  | Do you wash your hands before using gloves? | 1. Yes  2. No |  |  |
|  | Do you wash your hands after using gloves? | 1. Yes  2. No |  |  |
|  | Do you shorten your finger nails regularly? | 1. Yes  2. No |  |  |
|  | Do you wear hair cover/cape while handling or cooking food? | 1. Yes  2. No |  |  |
|  | Do you wash your hands after sneezing and touching your nose? | 1. Yes  2. No |  |  |

**Part-III- Questions on food safety knowledge**

| **S/N** | **Questions** | **Choice of response** | **Code** | **SKIP** |
| --- | --- | --- | --- | --- |
|  | Contaminated food always have some change in color, odor or taste | 1. Yes  2. No |  |  |
|  | Healthy people can cause illness by carrying germs to food | 1. Yes  2. No |  |  |
|  | Cooked foods do not have microbes | 1. Yes  2. No |  |  |
|  | Contact between raw and cooked foods contributes to food contamination | 1. Yes  2. No |  |  |
|  | Hand hygiene can prevent food contamination | 1. Yes  2. No |  |  |
|  | Food handler with a disease such as diarrhea, flue and sore throat poses a risk of food contamination | 1. Yes  2. No |  |  |
|  | Proper cleaning and sanitation of the utensils are very important in the food safety | 1. Yes  2. No |  |  |
|  | Use of jewels such as rings, watches, wearing in food handling cause food contamination | 1. Yes  2. No |  |  |
|  | Temperature of 5-60c^0^ is considered as danger zone in food safety | 1. Yes  2. No |  |  |

**Part IV: Questions on Basic sanitary facilities**

| S. no | Questions | Choices of response | Code | Skip |
| --- | --- | --- | --- | --- |
|  | Is there Liquid waste storage system | 1. Yes 2. No |  |  |
|  | Is there Functional kitchen? | 1.Yes  2.No |  |  |
|  | Is there Clean Utensil? | 1.Yes  2.No |  |  |
|  | Is there Shelf/ cupboard? | 1.Yes  2.No |  |  |
|  | Do you use three compartment dishwashing systems? | 1.Yes  2.No |  |  |
|  | Is there functional refrigerator in kitchen? | 1.Yes  2.No |  |  |
|  | Is there water storage equipment? | 1.Yes  2.No |  |  |

**Part IV- Questions on institutional factors**

| S/N | Questions | Choices of response | Code | Skip |
| --- | --- | --- | --- | --- |
|  | Have you ever attended food safety training | 1. Yes 2. No |  |  |
|  | How did you acquire the training | 1. Personally by training centers 2. By the food establishment 3. By health bureau 4. By cultural and tourism Bureau 5. Others specify ------ |  |  |
|  | Is there a food safety practice guideline? | 1. Yes 2. No |  |  |
|  | Is there sanitary inspection conducted recently? | 1. Yes 2. No |  |  |

**Part VI- Questions on Health related factors**

| S/N | Questions | Choices of response | Code | Skip |
| --- | --- | --- | --- | --- |
| 601 | Do you have medical checkup regularly? | 1.Yes  2.No |  |  |
| 602 | Do you take sick leave during illness? | 1.Yes  2.No |  |  |

**Thank You!**
